# Calibration of Traceable Solid Mock ^131^I Phantoms Used in an International SPECT Image Quantification Comparison

**DOI:** 10.6028/jres.118.017

**Published:** 2013-08-15

**Authors:** BE Zimmerman, L Pibida, LE King, DE Bergeron, JT Cessna, MM Mille

**Affiliations:** 1 National Institute of Standards and Technology, Gaithersburg, MD 20899; 2 Nuclear Engineering and Engineering Physics Program, Rensselaer Polytechnic Institute, Troy, NY 12180

**Keywords:** barium-133, iodine-131, SPECT, standards, traceability

## Abstract

The International Atomic Energy Agency (IAEA) has organized an international comparison to assess Single Photon Emission Computed Tomography (SPECT) image quantification capabilities in 12 countries. Iodine-131 was chosen as the radionuclide for the comparison because of its wide use around the world, but for logistical reasons solid ^133^Ba sources were used as a long-lived surrogate for ^131^I. For this study, we designed a set of solid cylindrical sources so that each site could have a set of phantoms (having nominal volumes of 2 mL, 4 mL, 6 mL, and 23 mL) with traceable activity calibrations so that the results could be properly compared. We also developed a technique using two different detection methods for individually calibrating the sources for ^133^Ba activity based on a National standard. This methodology allows for the activity calibration of each ^133^Ba source with a standard uncertainty on the activity of 1.4 % for the high-level 2-, 4-, and 6-mL sources and 1.7 % for the lower-level 23 mL cylinders. This level of uncertainty allows for these sources to be used for the intended comparison exercise, as well as in other SPECT image quantification studies.

## 1. Introduction

The International Atomic Energy Agency (IAEA) recently initiated a Cooperative Research Project (CRP) entitled “Development of Quantitative Nuclear Medicine Imaging for Patient Specific Dosimetry” as part of a larger program aimed at enhancing the practice of nuclear medicine physics in its Member States. One of the early goals of the CRP is to assess the global state of the accuracy and consistency of Single Photon Emission Computed Tomography (SPECT) image quantification, as evidenced by the performance of the institutions participating in the project during a series of comparison exercises. The 12 participants represent a cross-section of imaging experience worldwide, ranging from large research institutions in developed countries with decades of experience in SPECT imaging to clinical centers in developing countries that have limited imaging experience but which are now seeking to expand their capabilities to, for example, the use of imaging data to perform internal dosimetry treatment planning for nuclear medicine.

One of the goals of the CRP is to enhance and promote the use of image-based patient-specific dosimetry protocols in the IAEA Member States. To help achieve this, it was decided that an assessment of image quantification methodologies used in the participating sites should be performed and that this should take the form of a blind comparison study. The radionuclide and source configuration for the comparison were selected based on clinical relevance and familiarity to the participants.

Based on information provided by the participants regarding the type of instrumentation (i.e., activity calibrators and scanners) that they have available and the types of medical procedures that are performed in their centers, it was decided that ^131^I should be used as the test radionuclide for the comparison. Use of this radionuclide for diagnosis and therapy was noted for every one of the participating centers, for a variety of diseases. Moreover, this radionuclide was the one most often used by the participants, especially in the less developed countries, surpassing other commonly used medical radionuclides such as ^99m^Tc.

Iodine-131 has been used effectively for several decades as an adjuvant treatment following surgery against certain types of thyroid cancers, especially differentiated thyroid carcinoma [1–3]. More recently, targeted radiotherapy using agents, such as ^131^I-metaiodobenzylguanadine (^131^I-MIBG) and ^131^I-tositumomab (under the trade name Bexxar)^1^, have been used for treatment against neuroendricine tumors and non-Hodgkin’s lymphoma, respectively [4,5]. Many of the protocols for administration of these agents call for individualized treatment planning by using imaging data and internal dosimetry models.

As the quantity of interest in this comparison study was the accuracy with which the participants could determine the true activity concentration of the radioactive sources using SPECT imaging (with additional consistency check measurements made with a radionuclide calibrator), it was necessary for the activity content of the sources to be traceable to a single standard. The relatively short half-life (T_1/2_=8.0233(19) d) [6] of ^131^I and the large distances between the participating laboratories made it logistically impossible to prepare and calibrate phantoms of ^131^I and have them shipped to the participants with reasonable activity levels. For that reason, a longer lived surrogate radionuclide was required.

Barium-133 was chosen as the surrogate because of its long half-life of 10.540(6) a [7] and the fact that the most abundant γ-ray in the decay of ^133^Ba at 356 keV is similar in energy and emission probability to the most abundant γ-ray in the decay of ^131^I at 364 keV. This 2 % difference in energy is well within the energy resolution of the scanner detectors, thus no modifications to the ±10 % energy windows typically used for acquisition are necessary. One primary difference in the decay schemes, though, is the presence of a 637 keV γ-ray in ^131^I decay that is not found in ^133^Ba. Corrections for Compton scattering of this γ-ray into the acceptance energy window would normally have to be made for ^131^I, but this is not necessary for ^133^Ba.

For imaging, the differences in the level schemes between ^131^I and ^133^Ba are not expected to have a large effect beyond the difference in the emission probabilities of the primary γ-rays, which affects the conversion of the number of registered counts into activity values. For activity calibrator measurements, however, the differences are expected to be significant. For ^131^I, the average energy per decay from γ-rays alone is about 374 keV, while for ^133^Ba it is 360 keV. The presence of bremsstrahlung emitted from the slowing down of the β-particles emitted by ^131^I will also contribute to the response in the activity calibrator per decay. Consequently, for every becquerel of activity, ^131^I will produce a greater response in the instrument than ^133^Ba.

To minimize logistical problems associated with shipping liquid radioactive sources internationally, it was decided that the test objects for the comparison would be a set of 4 cylindrical phantom inserts, each containing a calibrated amount of ^133^Ba in epoxy. We had previously constructed and calibrated similar sources of this type, using ^68^Ge as the radionuclide [8,9]. In those studies, we found that it was extremely difficult to prepare the radioactive epoxy and dispense it into the empty cylinders with the level of homogeneity, reproducibility, and quantitative accuracy that would be acceptable for the sources to be used as calibration standards. We therefore adopted an approach of having the ^68^Ge epoxy sources manufactured by an experienced third party and calibrating them against well-characterized ^68^Ge solution standards in the same geometry. The success that was achieved in the case of ^68^Ge suggested that a similar approach could also be used to calibrate these ^133^Ba sources.

In order to maximize the sensitivity and be able to discern possible small effects in the participants’ measurement results, we set a goal of 1 % combined standard uncertainty on the total activity and activity concentrations of the individual sources. Although this was a somewhat arbitrary value, our past experience with ^68^Ge suggested that this was achievable.

This paper describes the methodology used to calibrate these solid epoxy-based ^133^Ba sources and gives the results of the experiments used to assay the sources used for the IAEA comparison.

## 2. Materials and Methods

### 2. 1 Epoxy Source Specifications and Construction

The sources were constructed of poly(methyl methacrylate) (PMMA) cylinders having a wall thickness of 1 mm and bottom thickness of 2 mm. The active length of the epoxy was nominally 3.8 cm and the inside diameters were varied to give approximate volumes of 2 mL, 4 mL, 6 mL, and 23 mL. These four source volumes constitute the set of sources that was sent to each participant. Each cylinder was fitted with a PMMA screw cap having a total length of 1 cm into which a 5 mm diameter threaded hole is blind tapped. The threaded hole accommodates a Plexiglas rod that is used to mount the cylinder to the bottom of a standard Jaczszak phantom. All the cylinders, caps, and rods were constructed in-house.

The empty cylinders and caps were weighed at NIST before being sent to RadQual, LLC (“RadQual”), who prepared the radioactive epoxy and filled the cylinders in a manner similar to that described by Zimmerman and Cessna [10]. For the 2 mL, 4 mL, and 6 mL cylinders, the nominal activity concentration of ^133^Ba in the epoxy was 200 kBq·g^−1^, while an activity concentration of 50 kBq·g^−1^ was chosen for the 23 mL sources to lower the total amount of activity being shipped to each participant. The cylinders were also weighed at RadQual before and after filling. After the epoxy was cured, the sources were sent to NIST, where final masses were obtained. The masses of added epoxy in each source reported by RadQual and NIST were in agreement to within about 0.2 %.

**Fig. 1:**
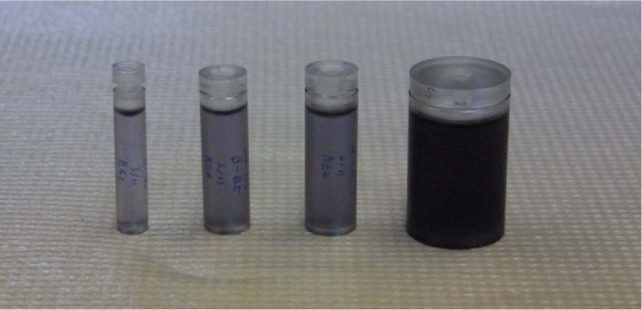
Photograph of a set of ^133^Ba epoxy sources prepared for and calibrated in this study. The sources appear dark due to a dye that was added to the epoxy during manufacturing as a visual indicator of uniform mixing.

After weighing at NIST, the sources were solvent-sealed and measured for total contained activity as described below. A set of the epoxy sources can be seen in Fig. 1.

### 2.2 Liquid-Filled Calibration Source Preparation

The instrumentation used to calibrate the epoxy sources, namely the NIST-maintained Vinten 671 secondary standard ionization chamber (VIC) and several high-purity germanium (HPGe) gamma-ray detectors, were calibrated by measurements against solution sources prepared using the identical PMMA cylinders used to construct the epoxy sources. The activity levels of the solutions used to fill the cylinders were intended to match those of the epoxy sources, namely 200 kBq·g^−1^ (“high-level”, HL) and 50 kBq·g^−1^ (“low-level”, LL), and both solutions had to be traceable to the same standard. Thus, it was necessary to prepare two stock solutions from a single calibrated master.

A master solution of ^133^Ba in 0.1 mol·L^−1^ HCl with an activity concentration of nominally 37 MBq·mL^−1^ was received from Eckert and Ziegler Isotope Products Laboratories (Valencia, CA). Approximately 3.3 g of this solution was added to about 27 mL of a carrier solution containing about 5 mmol·L^−1^ of BaCl_2_ in 1.2 mol·L^−1^ HCl to give a second solution containing about 4.4 MBq·g^−1^ of ^133^Ba (solution D1). Using an automated dispenser, 5 mL of solution D1 was transferred to each of four 5 mL NIST standard flame-sealed glass ampoules for measurement in the NIST Secondary Standard Ionization Chamber “A” (SSIC) [11]. The total mass of the transferred solution was determined by weighing the ampoules on a microbalance before and after filling.

Each of the four ampoules was measured 40 times in the SSIC, in 4 groups of 10 measurements, alternating with 5 groups of 10 measurements of a ^226^Ra reference source (RRS). Results were analyzed as a ratio of the response of the ampoule to the response of the RRS. After correction for background, the resulting ratio is used with a calibration factor, or K-value, defined as the activity of a given radionuclide that would produce the same response as the RRS, to give the total activity in ampoule. The K-value currently used for ^133^Ba was determined in 1983 using solutions calibrated by 4π β-γ anticoincidence counting. For the present experiments, the activity concentration (in Bq·g^−1^) was calculated from the activity found in the SSIC measurements and the measured mass of transferred solution.

Two additional gravimetric dilutions were performed with the solution from one of the ampoules containing solution D1 to give two solutions, D2 and D3, which were the HL and LL masters that were used to prepare the calibration sources for the 2 mL, 4 mL, and 6 mL cylinders (HL) and the 23 mL cylinders (LL). The activity concentrations of solutions D2 and D3 were calculated from the measured activity concentration of solution D1 and the respective gravimetric dilution factors.

The respective solutions were dispensed into the cylinders of each volume using an automated dispenser. The total mass of transferred solution was determined by weighing the cylinders on an analytical balance (readability 0.0001 g) before and after filling.

### 2.3 Full-Energy-Peak Efficiency Calibration of the HPGe Detectors

The liquid-filled cylindrical sources were used to determine the full-energy-peak efficiency of four coaxial high purity germanium (HPGe) detectors for each of the cylinder volumes [12]. The full-energy-peak efficiency measurements for two of the HPGe detectors were performed at a source-to-detector distance of approximately 90 cm on a top-mount geometry (referred to as G-detector and T-detector). The full-energy-peak efficiency measurements for the other two detectors were performed at a source-to-detector distance of approximately 1 m, one on a side-mount geometry (referred to as N-detector) and the other on a top-mount geometry (referred to as B-detector). At least three 1-day long spectra of the liquid-filled cylindrical sources were acquired for each volume and each detector geometry to determine each detectors’ full-energy-peak efficiency for the different source volumes (2 mL, 4 mL, 6 mL and 23 mL). The gamma-ray emission probabilities and half-life values used in the determination of the detectors’ full-energy-peak efficiencies were taken from Ref. [7]. The liquid-filled cylinder full-energy-peak efficiencies were used to determine the activities of the epoxy-filled cylinders. Full-energy-peak efficiencies were obtained from Eq. (1).

(1) $$\epsilon (Ε)=\frac{N(E)}{T\times M\times A\times P(E)}\prod_{i}C_{i}$$

where *N*(*E*) is the number of counts in the full-energy peak, *T* is the live time, *ε*(*E*) is the full-energy-peak efficiency, *P*(*E*) is the gamma-ray emission probability at the energy *E, A* is the source activity per unit mass, *M* is the mass of the sample and Π*C_i_* is the product of the correction factors, *C_i_*, applied to the measurement.

In addition to the decay correction for the ^133^Ba during the measurement time that was applied to both the liquid and epoxy sources, additional corrections were applied to account for geometrical differences between the liquid-filled cylinders and the epoxy-filled cylinders and the self-attenuation due to difference in the liquid and epoxy densities. Details of how these were derived are given in Sec. 2.4.1.

The uncertainty on the efficiency was obtained using uncertainty propagation, assuming that all measured quantities are independent. The uncertainty for the full-energy-peak efficiency, *ε(E)*, is given by

(2) $$u_{\varepsilon }=\sqrt{{\left(\frac{\partial \varepsilon }{\partial N}\right)}^{2}u_{N}^{2}+{\left(\frac{\partial \varepsilon }{\partial T}\right)}^{2}u_{T}^{2}+{\left(\frac{\partial \varepsilon }{\partial A}\right)}^{2}u_{A}^{2}+{\left(\frac{\partial \varepsilon }{\partial P}\right)}^{2}u_{P}^{2}+{\left(\frac{\partial \varepsilon }{\partial M}\right)}^{2}u_{M}^{2}+{\sum_{i}\left(\frac{\partial \varepsilon }{\partial C_{i}}\right)}^{2}u_{C_{i}}^{2}}$$

where *u*_N_, *u*_T_, *u*_A_, *u*_P_, *u*_C_*_i_*, and *u*_M_ are the uncertainties associated with the quantities *N*(*E*), *T*, *A*, *P*(*E*), *C_i_*, and *M*, respectively.

### 2.4 Determination of Calibration Factors for the VIC

Calibration factors for the VIC measurements were determined by measuring each liquid-filled cylinder three times over the course of about 14 days, with the HL cylinders (2 mL, 4 mL, and 6 mL) measured about 25 days after the preparation of D1 and the LL cylinders (23 mL) measured about 90 days after D1 was prepared. In order to ensure consistent placement of the source within the VIC, the sources were measured using a specially-constructed thin-walled plastic disc at the bottom of the source dipper. Each measurement in the VIC consisted of 1000 repeated measurements of the ionization current for each source, taken at two-second intervals. A total of 14 measurements of the background current were made, also consisting of 1000 current measurements at two-second intervals, as well as 3 separate measurements of the ionization current produced by a ^226^Ra reference source, consisting of 10 measurements taken 2 seconds apart. These data were used to determine calibration factors (in terms of pA·MBq^−1^) for liquids in each of the four cylinder volumes. The details of how these calibration factors were derived are given in Secs. 2.4.2 and 3.3.

### 2.5 Calibration of Epoxy Sources

Both detection techniques (HPGe gamma-ray spectrometry and measurement in the VIC) were used to determine the total ^133^Ba activities in the epoxy-filled cylinders. The use of multiple techniques not only gives confidence in the measurement results, but also gives insight into possible systematic biases or method-dependent measurement effects.

#### 2.5.1 HPGe Measurements

Because of time constraints, some of the source sets were required to be sent to participants before they could be measured in the HPGe systems. However, all sources were measured in the VIC.

For the HPGe measurements of the epoxy sources, the counting conditions were the same as for the calibration sources. The spectrum generated by each of the epoxy sources was acquired for approximately one day in each of the HPGe B-, G- and T-detectors in the top-mount geometry and three additional times in the HPGe N-detector in the side-mount geometry.

Due to differences in the solution fill-height, composition and density between the epoxy sources and the liquid-filled sources used to calibrate the HPGe detectors, several corrections to the full-energy-peak efficiency calibration measurements in the N-detector were made using analytical and Monte Carlo radiation transport methods. The Monte Carlo radiation transport calculations were performed using the Monte Carlo N-Particle eXtended (MCNPX) code version 2.5.0 [13]. The measurement geometry was modeled in the code and included the detector (crystal, crystal deadlayer, aluminum endcap and crystal mount cup), the lead collimator surrounding the detector, the surrounding air, and the ^133^Ba cylinders placed at the correct counting position. Each simulation involved randomly sampling more than 10^8^ monoenergetic photons within the cylindrical sources, with a new simulation being run for each of the 4 prominent gamma-ray lines of ^133^Ba. In each case, MCNPX’s F8 tally was used to calculate the full-energy peak efficiency from energy distribution of pulses created in the designated sensitive region of the crystal. The simulations were performed in coupled photon-electron transport mode (mode p e) using MCNPX’s standard physics models and default settings. The photon and electron cross-sections were taken from the default MCLIB04 [14] and EL03 tables, respectively.

The analytical corrections to account for the differences in source volume for the B-, G- and T-detector top-mount geometries were performed assuming the source can be replaced by a point source placed at the center of the solution height [15]. Therefore, the efficiency measurement was corrected using the distance inverse square law. To account for the difference in the epoxy composition, the attenuation coefficients for the epoxy were obtained using the Photon Cross Sections Data (XCOM) Database available from the National Institute of Standards and Technology (NIST) website [16].

Because of the way that the liquid filled sources were dispensed there was considerable variability in the filling volume of the liquid filled sources. Moreover, the liquid filling heights were systematically lower than the heights of the corresponding epoxy-filled sources. Calculations using MCNPX were performed to study the effects of the solution fill-height to obtain the corrections for the side-mount geometry measurements for the N-detector. The differences in the correction factors calculated for the 2 mL and 23 mL sources, which had the largest differences in filling volume between the liquid and epoxy (in the same cylinder volume) were 0.03 % and 0.15 %, respectively.

#### 2.5.2 VIC Measurements

For the VIC measurements, the counting conditions were the same as for the calibration sources, except that the measurements were made for all cylinders over the course of 27 days and that a total of 22 separate background measurements were made.

Because of composition and density differences between the epoxy sources and the liquid-filled sources used to calibrate the VIC, it was expected that different photon attenuation and bremsstrahlung production might affect the measurement results. Corrections to the calibration factors were made by Monte Carlo calculations using the EGSnrcMP package with the DOSRZnrc user code [17,18].

Using the methods and geometry description previously used for ^68^Ge [9], the relative response of the VIC to ^133^Ba contained in each of the four cylinder volumes was simulated for both the solution and epoxy sources. The active lengths of the radioactive ^133^Ba in each source were calculated based on the average values of the actual filling volumes, calculated from the masses and the known densities, and the measured inside diameters for each volume cylinder. Input photon spectral data were taken from Bé, et al. [7] and a total of 10^8^ initial events were randomly sampled from the spectrum for each simulation. The results of these calculations and the determination of the final correction factor values are discussed in Sec. 3.3.

## 3. Results

All evaluation of measurement uncertainties throughout this work follow accepted conventions used by the NIST Radioactivity Group and are in accordance with those recommended by the principal metrology organizations [19]. All individual uncertainty components are given as estimated experimental standard deviations (or standard deviations of the mean, if appropriate), or quantities assumed to correspond to standard deviations regardless of the method used to evaluate their magnitude. Unless explicitly stated, all uncertainties cited in this paper are “standard uncertainties”, corresponding to one uncertainty interval. In accordance with NIST policy [20], the combined standard uncertainty is calculated by combining the individual uncertainty components in quadrature.

### 3.1 Determination of Activities in the Solution-Filled Calibration Sources

From the measurements in the NIST SSIC of the four ampoules containing solution D1, an average value of (4.45 ± 0.03) × 10^6^ Bq·g^−1^ was obtained for the activity concentration of that solution at a reference time of 12:00 EST 14 January, 2011. Applying the mass-determined dilution factors, activity concentrations of (1.886 ± 0.012) × 10^5^ Bq·g^−1^ and (5.483 ± 0.036) × 10^4^ Bq·g^−1^ were obtained for the HL and LL solutions, respectively, at the same reference time. The uncertainties on the above values are calculated from the quadratic addition of uncertainty components due to the ^133^Ba K-value for the SSIC (0.66 %), decay correction and ^133^Ba half-life (0.015 %), the standard deviation of the 40 repeated measurements made on each of the ampoules (0.01 %), and the standard deviation of the measured activity concentrations for the four D1 ampoules (0.051 %). An additional uncertainty component due to the uncertainty in the dilution factors (0.1 %) was included in the calculations of the uncertainty for the HL and LL solutions.

### 3.2 HPGe Measurements

**Fig. 2:**
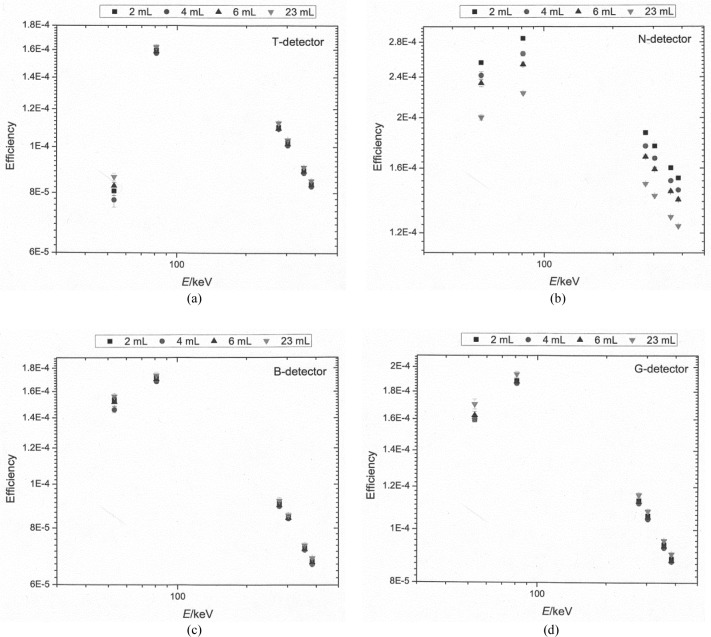
Full-energy-peak efficiencies for the four HPGe detectors used in the measurements of the liquid-filled vials. The uncertainty bars correspond to a single uncertainty interval (k = 1) calculated as described in Sec. 3.2.

The measured full-energy-peak efficiencies for the liquid-filled sources are shown graphically in Fig. 2. The uncertainty bars on the plot represent standard uncertainties as obtained using Eq. (2). The correction factors that were applied to the measured efficiency values (for each HPGe detector) to account for the geometrical differences (i.e., differences in solution fill-height in the cylinder) between the liquid-filled cylinders and the epoxy-filled cylinders, and the self-attenuation due to difference in the liquid and epoxy densities are listed in Tables 1 through 4. Although the full-energy-peak efficiencies were calculated over the entire energy ranges shown in Fig. 2, only the decay energies given in Tables 1–4 were used in the actual determination of the ^133^Ba activity.

The average activities for the epoxy sources of a given volume, as determined from the HPGe measurements, were obtained from combining the values of the 276.4 keV, 302.9 keV, 356.0 keV and 383.8 keV gamma-ray lines. The uncertainties on each source activity were calculated from the quadratic addition of the uncertainties due to the ^133^Ba standard solution calibration (0.66 %), measurement repeatability (standard deviation of the repeated measurements in the same HPGe detector for each source, 0.2 % to 0.4 %), decay correction (0.0006 % to 0.001 %, depending on when measurements were made), gamma-ray emission probabilities (0.3 % to 0.7 %), sample counting plus peak fitting (0.1 % to 0.3 %), sample geometry (standard deviation of the repeated measurements in the different HPGe detectors for each source, 0.02 % to 0.2 %), self-attenuation due to difference in the liquid and epoxy densities (0.2 % to 0.8 %), and differences in solution height for different cylinder fill-height (0.8 % to2 %, depending on the geometry).

**Fig. 3:**
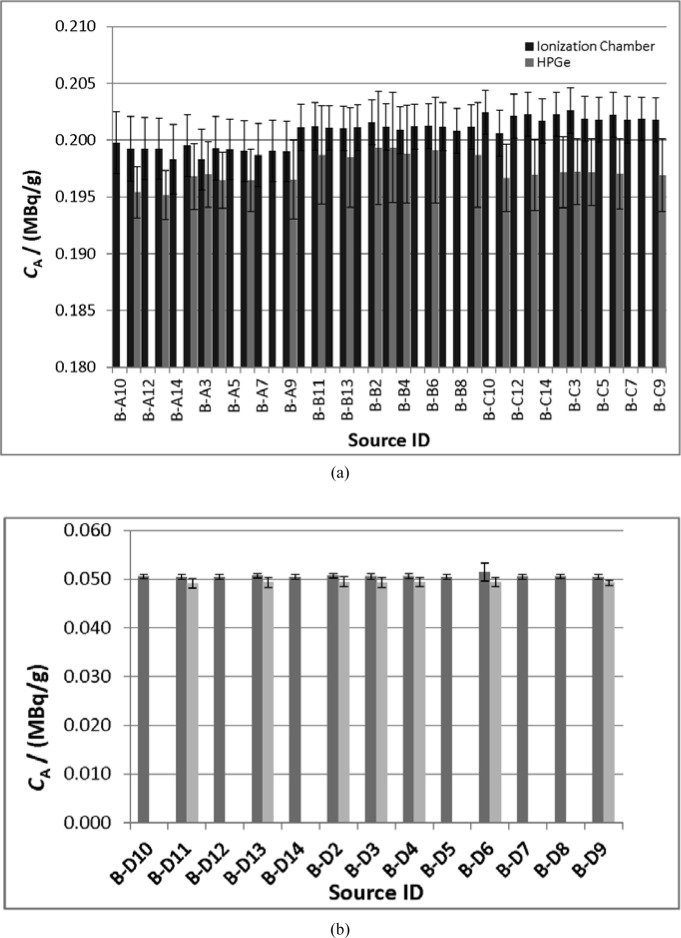
Plot of measured activity concentrations of ^133^Ba in epoxy for the “A” (2 mL), “B” (4 mL), and “C” (6 mL) cylinders series (a) and “D” (23 mL) series (b), as determined from the NIST Vinten 671 ionization chamber (VIC, dark bars) and the NIST HPGe detectors (light bars). The uncertainty bars correspond to a single (k = 1) uncertainty interval and were calculated as described in Secs. 3.2 and 3.3. Some source sets were sent to the comparison participants before they could be measured on the HPGE systems, therefore those values are not available.

The measured activity concentrations for each source, along with their uncertainties, are given in Fig. 3.

### 3.3 VIC Measurements

**Fig. 4:**
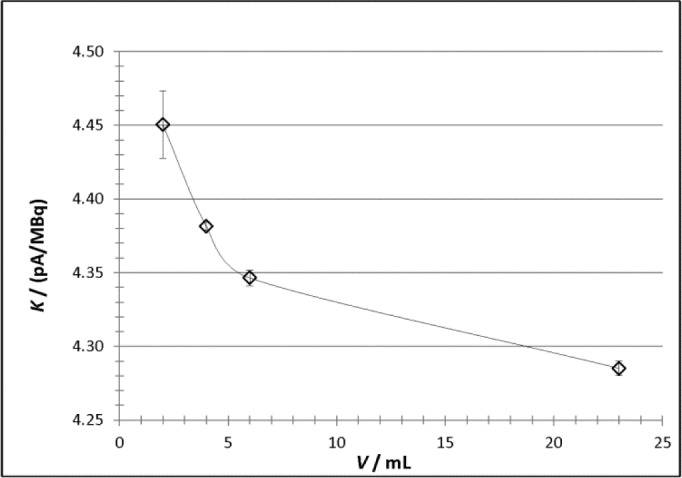
Experimentally-determined calibration factors, *K*, as a function of source volume for the NIST Vinten 671 ionization chamber (VIC) for the solution-filled ^133^Ba cylinders used in this study. The uncertainty bars correspond to a single uncertainty interval (*k* = 1) calculated as described in Sec. 3.3. The line through the data is intended only to guide the eye.

The calibration factors measured for each of the solution sources are shown graphically in Fig. 4. The uncertainty bars on the plot represent standard uncertainties and are equal to the combined standard uncertainties calculated from the quadratic combination of the uncertainty components presented in Table 5. The larger uncertainty on the result for the 2 mL cylinders is due primarily to the much lower current (relative to background) measured for those sources as compared to the others. For those sources, the current was only about 50 times that of background while the signal-to-background ratios for the other volumes were 2 to 5 times higher.

**Fig. 5:**
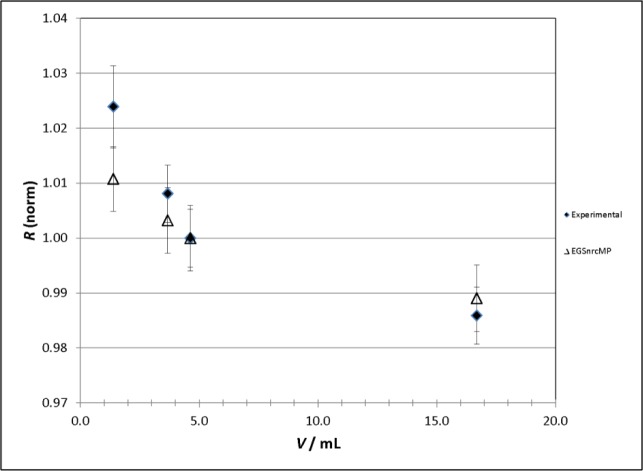
Plot of experimental and theoretically calculated relative response for ^133^Ba solution filled cylinders in the NIST Vinten 671 ionization chamber (VIC). For the experimental data (solid diamonds), the relative response, *R*(norm), is defined as the ratio of the average measured ionization current per unit activity (in A/Bq) for all sources in a particular volume set to that of the average measured ionization current for the sources having a nominal volume of 6 mL. For the theoretical data (open triangles), the relative response is given as the ratio of the dose per particle calculated from the DOSRZnrc simulations for each volume to that of the dose per particle calculated for the source having a nominal volume of 6 mL. All uncertainty bars correspond to single (*k* = 1) uncertainty intervals and their method of evaluation is described in Sec. 3.3.

The VIC calibration factors for the solution filled cylinders, normalized to the value at 6 mL, are plotted along with the response as calculated from the DOSRZnrc simulations, also relative to the value at 6 mL, in Fig. 5. Excellent agreement between the experimental and theoretical response is obtained for the 4 mL, 6 mL, and 23 mL cylinders, while the agreement at 2 mL can be considered reasonable, given the large uncertainties associated with the experimental measurement.

**Fig. 6:**
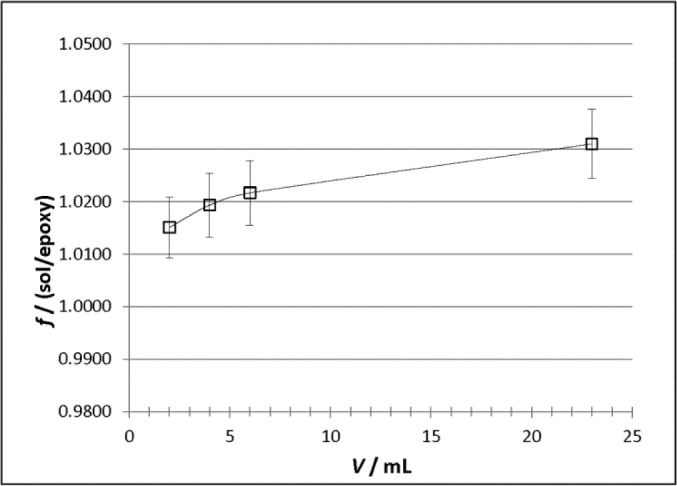
Calculated correction factors, *f*, as a function of source volume for the NIST Vinten 671 ionization chamber (VIC) for the epoxy ^133^Ba cylinders used in this study to account for attenuation differences between the solution and epoxy-filled cylinder sources. The uncertainty bars correspond to a single uncertainty interval (*k* = 1) calculated as described in Sec. 3.3. The line through the data is intended only to guide the eye.

The good agreement in the theoretical and experimental relative responses of the solution sources gives us confidence in the calculated correction factors that were subsequently used to account for differences in attenuation between the epoxy and the ^133^Ba standard solution. These correction factors were calculated for each cylinder volume as *f*_v_ = *D*_sol,v_ / *D*_Ep, v_, where *D*_sol,v_ and *D*_Ep,v_ are the absorbed doses in the ionization chamber gas for the ^133^Ba solution and epoxy, respectively, calculated from the Monte Carlo simulations using the average dispensed volume in the cylinders of each source type. These volumes were calculated from the dispensed masses and the densities for the solution and epoxy. The uncertainty for each *f*_v_ was calculated by summing the uncertainty reported by DOSRZnrc for each dose region for each source to give an uncertainty on *D*_sol,v_ and *D*_Ep,v_, then these were added in quadrature to give an uncertainty on the ratio. The resulting correction factors, along with their uncertainties, are shown in Fig. 6.

The ^133^Ba activity in each epoxy source was calculated using the average measured ionization chamber current, the calibration factor, and the respective correction factor appropriate for each volume. The uncertainties on each source were calculated from the quadratic addition of the uncertainties due to the calibration factors (see Table 5), measurement repeatability (standard deviation of three ionization chamber measurements for each source, 0.1 % to 0.4 %), decay correction (0.01 % to 0.4 %, depending on when measurements were made), and background variability (0.1 % to 0.3 %, depending on ionization current, and thus total activity in the source).

The measured activity concentrations for each source, along with their uncertainties, are given in Fig. 3, along with the HPGe measurement results.

## 4. Discussion

Both of the techniques used in this study are able to provide the ^133^Ba activity values in the epoxy-filled cylinders with a combined standard uncertainty on the activity of any individual source of about 1 %. The agreement in activity values for each source is within the experimental uncertainties for the two techniques. Looking carefully at the data in Fig. 3, however, it is evident that the HPGe-derived activity values are systematically lower than the VIC value for the same source.

This effect is also evident when the cylinders of each volume set are considered as a group, as seen in Table 6. When evaluated in this way, the magnitudes of the differences were 1.1 %, 1.1 %, 2.4 % and 1.9 % for the 2 mL, 4 mL, 6 mL, and 23 mL sources, respectively. This is about the same as the individual uncertainties (1 %), except for the 6 mL sources, for which the difference is somewhat higher, but still within the combined uncertainties for the respective techniques.

This systematic effect is most likely a result of the different approaches taken to making the corrections for differences in photon attenuation between the liquid-filled calibration sources and the epoxy-filled cylinders. In both cases, there is a strong reliance on Monte Carlo simulation or analytical methods to determine the correction factors. Despite the large amount of literature that can be found validating the various Monte Carlo radiation transport packages that are available, the fact that each package uses its own cross-section databases and implements radiation transport in a slightly different way makes this type of systematic difference almost inevitable. Other factors, such as the accuracy and level of detail of the geometry model used in the simulation, can also play a role. While it would have perhaps been more desirable to use the same package to simulate both detection techniques, the choices of which package to use essentially reflected the users’ familiarity with the respective code package and facility in developing a geometry model. In the case of the VIC model, for example, the same basic geometry description has been successfully applied to previous studies [10] and is routinely used in our laboratory as a verification of experimental results.

Since we cannot definitively attribute the systematic differences between the two techniques solely to the self-attenuation corrections, we have no justification for judging either of the methods as being superior to the other. Therefore, we should consider this difference to be an additional component of uncertainty in trying to arrive at a single value for the activity or activity concentration of the ^133^Ba in each source. Several approaches are available that allow for the estimation of between-laboratory or between-method uncertainties [21–25], with most of them relying on some sort of maximum likelihood analysis. The Consensus Mean module of the DATAPLOT program [26] uses several approaches in addition to those referenced above to solve this problem and reports the consensus mean and magnitude of the between-laboratory variance for each technique.

Because all the 2 mL, 4 mL, and 6 mL cylinders were prepared from the same batch of epoxy within a short amount of time, it is a valid assumption that the ^133^Ba activity concentration is the same in those samples. Therefore, all the results for the VIC were combined into one group of 39 measurements and the HPGe data were combined to form a second group of 21 measurements. These two groups were then analyzed using DATAPLOT to arrive at a single consensus value and an estimate of the between-method variance.

The means calculated with 6 different implementations of the consensus mean approach were all reported to be 0.199 MBq·g^−1^ with a relative inter-method standard deviation of 0.59 %. Taking a mean standard uncertainty of 1.33 % for the measurement of the ^133^Ba activity concentration on a single source that was calculated from the 60 determinations of the activity concentration from the two methods, and adding the inter-method standard deviation in quadrature, we obtain a new combined standard uncertainty of 1.46 % on the ^133^Ba activity concentration in those cylinders. Therefore, the final value that will be used for the activity concentration of the ^133^Ba in the 2 mL, 4 mL, and 6 mL cylinders is (0.199 ± 0.003) MBq·g^−1^ at the established reference time, assuming a standard (*k* = 1) uncertainty.

Applying the same approach to the measurement of the activity concentration in the 23 mL cylinders, we calculate a consensus mean value of 0.050 MBq·g^−1^ for the activity concentration and a relative inter-method standard deviation of 1.00 %. Combining the inter-method standard deviation in quadrature with an average combined standard uncertainty of 1.43 % for the activity concentration determination on a single source, a final value of (0.0503 ± 0.0009) MBq·g^−1^, where the new relative combined standard uncertainty is 1.74 %.

Although the approach that we have taken in arriving at single values for the activity concentrations for the two groups of sources results in much larger uncertainties than were obtained from the independent measurements with two different techniques taken on their own, it reflects a much more realistic view of how well the measurements of these types of sources can be made. Even at this level, however, the uncertainty on the calibrated ^133^Ba activity concentration is still much lower, by at least an order of magnitude than any of the uncertainties that are expected from the inter-laboratory imaging comparison.

## 5. Conclusion

We have developed a method to calibrate a series of cylinder sources containing ^133^Ba-spiked epoxy that are being used as test standards for an IAEA-sponsored inter-laboratory comparison for SPECT image quantification. The method involved quantification of the contained activity in the cylinders using two detection techniques, namely measurement in a re-entrant ionization chamber and gamma-ray spectrometry using HPGe detectors. While each technique taken on its own is able to provide a value for the ^133^Ba activity concentration in a single source with a relative combined uncertainty of about 1 %, a systematic difference of 1.1 % to 2.4 % between the results obtained with the two methods was observed. By considering the two data sets together and assuming that the overall uncertainty consisted of a within-method uncertainty as well as a between-method uncertainty, we assigned average ^133^Ba activity concentration values to the “high” and “low” level ^133^Ba epoxies studied, with relative combined uncertainties of 1.4 % and 1.7 %, respectively.

Although the uncertainties on the activity concentration values are somewhat higher than our original goal of 1 %, this is still much lower than the uncertainties expected in the imaging comparison data. These sources should be appropriate as test standards in the investigation of other aspects of SPECT image quantification.

## Figures and Tables

**Table 1:** HPGe full-energy-peak efficiency measurements and applied correction factors for the B-detector.

Source type	Gamma-ray peak energy (keV)	Efficiency	Efficiency uncertainty (%)	Self-attenuation correction factor	Solution fill height correction factor
2 mL cylinders	276.3989	9.072E-05	0.567	0.915	0.986
	302.8508	8.496E-05	0.553	0.918	0.986
	356.0129	7.266E-05	0.264	0.923	0.986
	383.8485	6.769E-05	0.391	0.926	0.986
4 mL cylinders	276.3989	8.943E-05	0.765	0.915	0.986
	302.8508	8.398E-05	0.753	0.918	0.986
	356.0129	7.160E-05	0.376	0.923	0.986
	383.8485	6.658E-05	0.621	0.926	0.986
6 mL cylinders	276.3989	8.999E-05	0.588	0.915	0.986
	302.8508	8.429E-05	0.877	0.918	0.986
	356.0129	7.191E-05	0.307	0.923	0.986
	383.8485	6.730E-05	0.461	0.926	0.986
23 mL cylinders	276.3989	9.193E-05	1.486	0.915	0.986
	302.8508	8.542E-05	1.193	0.918	0.986
	356.0129	7.328E-05	1.254	0.923	0.986
	383.8485	6.868E-05	0.976	0.926	0.986

**Table 2:** HPGe full-energy-peak efficiency measurements and applied correction factors for the G-detector.

Source type	Gamma-ray peak energy (keV)	Efficiency	Efficiency uncertainty (%)	Self-attenuation correction factor	Solution fill height correction factor
2 mL cylinders	276.3989	1.133E-04	0.475	0.915	0.986
	302.8508	1.061E-04	0.460	0.918	0.986
	356.0129	9.398E-05	0.240	0.923	0.986
	383.8485	8.855E-05	0.389	0.926	0.986
4 mL cylinders	276.3989	1.120E-04	0.540	0.915	0.986
	302.8508	1.046E-04	0.529	0.918	0.986
	356.0129	9.271E-05	0.282	0.923	0.986
	383.8485	8.751E-05	0.450	0.926	0.986
6 mL cylinders	276.3989	1.129E-04	0.433	0.915	0.986
	302.8508	1.056E-04	0.440	0.918	0.986
	356.0129	9.337E-05	0.223	0.923	0.986
	383.8485	8.792E-05	0.359	0.926	0.986
23 mL cylinders	276.3989	1.160E-04	0.769	0.915	0.986
	302.8508	1.083E-04	0.564	0.918	0.986
	356.0129	9.559E-05	0.696	0.923	0.986
	383.8485	9.031E-05	0.398	0.926	0.986

**Table 3:** HPGe full-energy-peak efficiency measurements and applied correction factors for the T-detector

Source type	Gamma-ray peak energy (keV)	Efficiency	Efficiency uncertainty (%)	Self-attenuation correction factor	Solution fill height correction factor
2 mL cylinders	276.3989	1.100E-04	0.616	0.915	0.985
	302.8508	1.026E-04	0.875	0.918	0.985
	356.0129	8.928E-05	0.309	0.923	0.985
	383.8485	8.363E-05	0.553	0.926	0.985
4 mL cylinders	276.3989	1.092E-04	0.565	0.915	0.985
	302.8508	1.008E-04	0.652	0.918	0.985
	356.0129	8.827E-05	0.270	0.923	0.985
	383.8485	8.265E-05	0.438	0.926	0.985
6 mL cylinders	276.3989	1.094E-04	0.452	0.915	0.985
	302.8508	1.016E-04	0.449	0.918	0.985
	356.0129	8.902E-05	0.242	0.923	0.985
	383.8485	8.325E-05	0.362	0.926	0.985
23 mL cylinders	276.3989	1.124E-04	1.551	0.915	0.985
	302.8508	1.035E-04	1.262	0.918	0.985
	356.0129	9.074E-05	1.180	0.923	0.985
	383.8485	8.498E-05	1.168	0.926	0.985

**Table 4:** HPGe full-energy-peak efficiency measurements and applied correction factors for the N-detector

Source type	Gamma-ray peak energy (keV)	Efficiency	Efficiency uncertainty (%)	Self-attenuation and solution fill height correction factor
2 mL cylinders	276.3989	1.873E-04	0.459	0.9970
	302.8508	1.765E-04	0.447	0.9971
	356.0129	1.602E-04	0.239	0.9976
	383.8485	1.532E-04	0.362	0.9979
4 mL cylinders	276.3989	1.765E-04	0.435	0.9962
	302.8508	1.672E-04	0.586	0.9965
	356.0129	1.513E-04	0.217	0.9963
	383.8485	1.451E-04	0.489	0.9966
6 mL cylinders	276.3989	1.683E-04	0.367	0.9956
	302.8508	1.592E-04	0.371	0.9954
	356.0129	1.443E-04	0.362	0.9958
	383.8485	1.391E-04	0.441	0.9959
23 mL cylinders	276.3989	1.491E-04	0.432	0.9894
	302.8508	1.414E-04	0.435	0.9893
	356.0129	1.288E-04	0.219	0.9917
	383.8485	1.237E-04	0.355	0.9921

**Table 5:** Uncertainty components for the determination of calibration factors in the VIC for ^133^Ba in the solution-filled cylinders used in this study. Values are provided for each volume series (A-D) separately.

Component	Eval. type; Comment	*u,_iA_*, %	*u,_iB_*, %	*u,_iC_*, %	*u,_iD_*, %
^133^Ba activity	B; Standard uncertainty on calibration of ^133^Ba solution, includes 0.05 % for dilution factor to D2.	0.66	0.66	0.66	0.66
Measurement repeatability	A; Standard deviation of the mean on 1000 measurements of the NPL chamber ionization current; average of 9 measurements	0.54	0.24	0.19	0.2
Measurement reproducibility	A; standard deviation on the average K_act_ determined for 3 sources of the same volume (2 sources for 4 mL)	0.51	0.12	0.12	0.12
Decay correction	B; uncertainty on activity due to 0.06 % uncertainty in ^133^Ba half-life	2.4×10^4^	3.1×10^4^	9.3×10^4^	8.3×10^4^
Calculation of correction factor for solution-epoxy response	B; uncertainty in ratio of simulated IC responses between solution and epoxy sources	0.57	0.59	0.60	0.64
Background	A; standard deviation on 10 measurements of the background ionization current	0.58	0.22	0.18	0.18
$u_{c}={\left(\sum u_{i}^{2}\right)}^{1/2}$		1.28	0.95	0.94	0.96

**Table 6:** Average activity concentrations for the 133Ba epoxy-filled cylinders sources of a given volume, along with the percent difference between the averages, as determined from HPGe and VIC measurements. The uncertainties here are the combined uncertainties of the average standard uncertainty for each source combined with the standard deviation on the sources within each set. As described in the text, the final activity concentration value assigned to the 2 mL, 4 mL, and 6 mL series was (0.199 ± 0.003) MBq·g^−1^, while that for the 23 mL sources was (0.0503 ± 0.0009) MBq·g^−1^.

Series	*C*_A_, MBq/g, HPGe	*C*_A_, MBq/g, VIC	Δ
2 mL	0.196(4)	0.198(2)	1.1 %
4 mL	0.199(5)	0.201(2)	1.1 %
6 mL	0.197(3)	0.201(2)	2.4 %
23 mL	0.0493(5)	0.0502(5)	1.9 %

## Footnotes

1 Certain commercial equipment, instruments, or materials are identified in this paper to foster understanding. Such identification does not imply recommendation by the National Institute of Standards and Technology, nor does it imply that the materials or equipment identified are necessarily the best available for the purpose.
